# Where Do Neurologists Look When Viewing Brain CT Images? An Eye-Tracking Study Involving Stroke Cases

**DOI:** 10.1371/journal.pone.0028928

**Published:** 2011-12-12

**Authors:** Hideyuki Matsumoto, Yasuo Terao, Akihiro Yugeta, Hideki Fukuda, Masaki Emoto, Toshiaki Furubayashi, Tomoko Okano, Ritsuko Hanajima, Yoshikazu Ugawa

**Affiliations:** 1 Department of Neurology, University of Tokyo, Tokyo, Japan; 2 Segawa Neurological Clinic for Children, Tokyo, Japan; 3 Interfaculty Initiative in Information Studies, University of Tokyo, Tokyo, Japan; 4 Department of Neurology, School of Medicine, Fukushima Medical University, Fukushima, Japan; University Of Cambridge, United Kingdom

## Abstract

The aim of this study was to investigate where neurologists look when they view brain computed tomography (CT) images and to evaluate how they deploy their visual attention by comparing their gaze distribution with saliency maps. Brain CT images showing cerebrovascular accidents were presented to 12 neurologists and 12 control subjects. The subjects' ocular fixation positions were recorded using an eye-tracking device (Eyelink 1000). Heat maps were created based on the eye-fixation patterns of each group and compared between the two groups. The heat maps revealed that the areas on which control subjects frequently fixated often coincided with areas identified as outstanding in saliency maps, while the areas on which neurologists frequently fixated often did not. Dwell time in regions of interest (ROI) was likewise compared between the two groups, revealing that, although dwell time on large lesions was not different between the two groups, dwell time in clinically important areas with low salience was longer in neurologists than in controls. Therefore it appears that neurologists intentionally scan clinically important areas when reading brain CT images showing cerebrovascular accidents. Both neurologists and control subjects used the “bottom-up salience” form of visual attention, although the neurologists more effectively used the “top-down instruction” form.

## Introduction

In clinical practice, neurologists often use brain computed tomography (CT) images to detect lesions in patients. During the visual search for a lesion, neurologists' eyes move in various directions in the course of examining each brain CT image. To date, precisely what they are looking at while examining these images, and what kinds of visual attention they use during this process, remains unknown.

Visual attention is roughly divided into two information-processing mechanisms: “top-down instruction” and “bottom-up salience” [1]–[4]. Top-down instruction indicates that attention is allocated to an object in a goal-oriented manner, with various types of goals depending on the circumstances. In contrast, bottom-up salience indicates that attention is captured by a visually conspicuous object, irrespective of the subject's intention. These two information-processing mechanisms usually overlap each other [1]. Here, we used an eye-tracking device to investigate the patterns of visual attention involved in searching for lesions in brain CT images. This device allows us to create heat maps, a means of objectively visualizing the distribution of a subject's gaze over an image [5]. The eye-tracking device also enables us to sequentially record the positions where the eyes are fixed in order to elucidate what the observers are looking at and when. We can then determine the type or types of visual attention taking place in the brain by comparing our eye-tracking data with saliency maps of the CT images.

Saliency mapping is a conceptually simple computational model of focal visual attention that simulates bottom-up, image-based attentional deployment, accurately identifying the objectively outstanding areas in an image [6]. If the areas in an image that are identified as outstanding through saliency mapping overlap with the areas on which a subject's gaze is frequently fixated in eye-tracking analysis, this indicates that the subject's attention is being captured by visually salient objects, i.e., that the subject is engaging in “bottom-up salience” [7]. Analysis using saliency maps has so far been limited to images of visual scenes [6], [8]–[10], and has not previously been applied to radiographic images.

The aim of this paper is to investigate what neurologists look at when they view brain CT images of patients who have suffered cerebrovascular accident and to evaluate the type of visual attention that they use in the interpretation of these images. First, we presented several brain CT images to neurologists and control subjects and recorded their eye-fixation positions using an eye-tracking device. Next, we identified the region of interest (ROI) in each image and compared the dwell time of eye-fixation at the ROI between the two subject groups. Third, we sought to determine whether the neurologists were more likely to notice clinically important areas, some of which were visually non-salient, which control subjects failed to detect. For this purpose, we defined clinically important areas as those which could be associated with the diagnosis, cause, prognosis, or treatment for stroke.

## Methods

### Subjects

A total of 24 subjects, including 12 neurologists and 12 control subjects, all with normal or corrected-to-normal (via contact lenses) vision participated in this study. All of the neurologists had experience in stroke care and in reading brain CT images. The average length of their careers in neurology to date was 7.1 years (range, 3–19 years). The controls consisted of other medical practitioners (nurses, medical technologists, psychologists, and medical students), all of whom had some knowledge about the brain but had not received any formal training on reading brain CT images. The two groups of subjects were age-matched (mean age of neurologists ± SD: 33.0±6.3 years [range, 26–43 years[; controls: 33.2±10.5 years [range, 22–59 years]).

Written informed consent to participate in this study was obtained from all subjects. The protocol was approved by the Ethics Committee of The University of Tokyo, and was conducted in accordance with the ethical standards of the Declaration of Helsinki.

### Eye-tracking device

Subjects were seated, and a steady head position was maintained with the aid of chin and forehead rests. The EyeLink 1000 system (SR Research, Mississauga, Ontario, Canada) was used to acquire eye-position data at a sampling rate of 1000 Hz. Gaze data were recorded from the right eye. Tasks were created using SR Research Experiment Builder version 1.5.58, and images were presented on a Dell E173FPb monitor at 60 Hz. The distance between the screen and the subject was a constant 50 cm, so that each image subtended a total visual angle of 38×30°, with 0.85 cm on the screen corresponding to approximately 1° of visual angle. Prior to the experiments, a nine-point calibration procedure was performed for each subject to map the eye-fixation position to screen coordinates. The calibration was considered to be valid if the maximum spatial error was less than 1° and the average error was less than 0.5°.

Every time the subject pushed a button connected to the eye-tracking device, a stimulus image appeared on the monitor and remained there for 20 seconds. The images appeared in a randomized order. The subject was instructed to interpret each presented brain CT image and to give a radiographic diagnosis with regard to cerebrovascular accident (Where are the lesions? What do you think should be the radiographic diagnosis?). One normal and five abnormal brain CT images were presented: 1) normal brain, 2) cerebral hemorrhage from putamen, 3) cerebral infarction due to embolism, 4) lacunar infarction, 5) hyperacute cerebral infarction with old infarctions, and 6) subarachnoid hemorrhage with acute subdural hemorrhage. The rates of correct radiographic diagnosis given by neurologists and controls were as follows: image 1: neurologists 83.3% (10/12), controls 16.7% (2/12), p = 0.014; image 2: neurologists 100.0% (12/12), controls 0.0% (0/12), p<0.001; image 3: neurologists 100.0% (12/12), controls 0.0% (0/12), p<0.001; image 4: neurologists 100.0% (12/12), controls 0.0% (0/12), p<0.001; image 5: neurologists 50.0% (6/12), controls 0.0% (0/12), p = 0.006; image 6: neurologists 58.3% (7/12), controls 0.0% (0/12), p = 0.002 (Mann-Whitney's U test). None of the controls were able to give a correct diagnosis for any of the abnormal brain CT images showing cerebrovascular accident (images 2–6). All of the neurologists gave correct diagnoses for images 2–4, although only about half of the neurologists were able to do so for images 5 and 6. In particular, the masked lesion in image 5 seemed to be the most difficult image to diagnose because of the low contrast of hyperacute cerebral infarction [11].

### Saliency mapping

Saliency maps were also created from the CT images using MATLAB 2009a and MATLAB implementation software [12]. This MATLAB implementation software was designed on the basis of a bottom-up visual saliency model known as graph-based visual salience [13]. The saliency mapping technique used in the present study can successfully predict human eye-fixation patterns more successfully than the classical algorithms of Itti et al. did [14]. The accuracy of its predictions can be confirmed through comparison with data on human eye-fixation patterns while viewing the same scene [6]. In the CT images used in the present study, the sharp contrast between the cranium and the image background resulted in the contour of the cranium being detected as the most salient region in the image. In practice, however, subjects never gazed at the rim of the cranium or the area outside it (see Results). Therefore, before saliency maps were generated, the cranium and the area outside it on original CT images were filled with the average color of the brain parenchyma in order to remove the strong contrast along the rim of the cranium.

### Heat mapping

**Figure 1:**
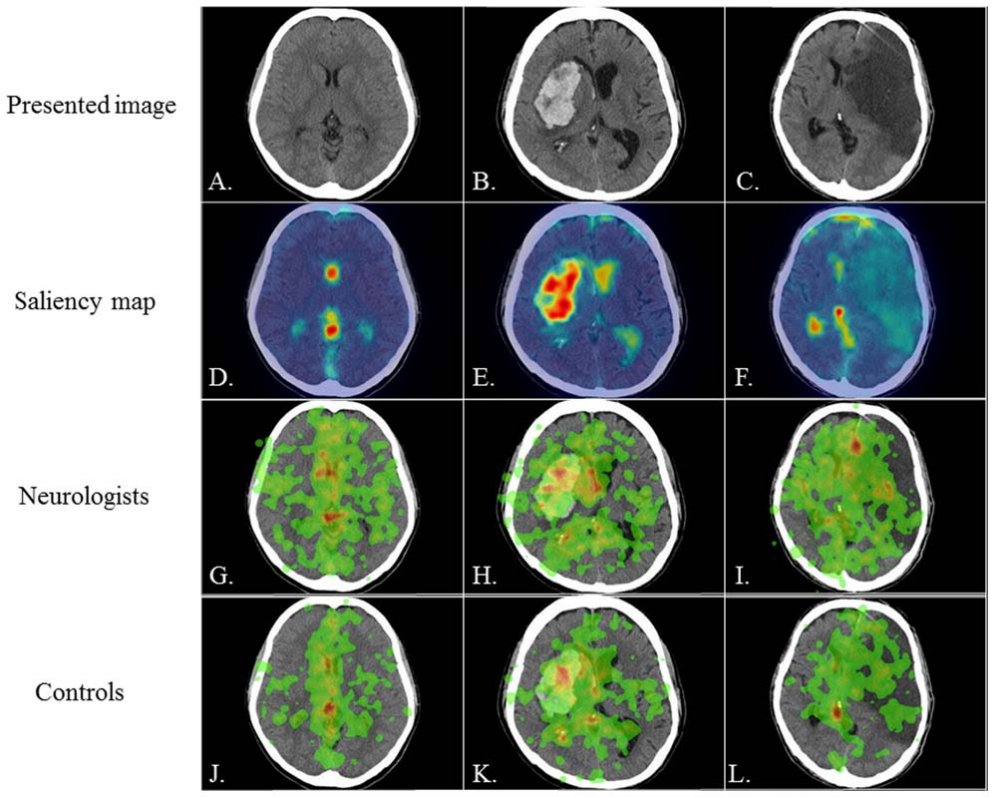
The presented images (images 1–3), saliency maps, and heat maps. A–C: the three CT images presented to subjects, D–F: saliency maps, G–I: heat maps in neurologists, J–L: heat maps in controls. Presented CT images are the normal brain (A: image 1), cerebral hemorrhage from the putamen (B: image 2), and cerebral infarction due to embolism (C: image 3). Saliency maps reveal that the most outstanding areas are the ventricles and cistern (D), the large hemorrhagic area (E), and the region of physiological calcification (F). Heat maps in neurologists and controls reveal that the most frequently fixated areas are similar between the two groups in images 1 and 2 but not in image 3; specifically, they are the ventricles and cistern in image 1 (G, J), the large hemorrhagic area in image 2 (H, K), and the ACA infarction area for neurologists and the region of physiological calcification for controls in image 3 (I, L).

**Figure 2:**
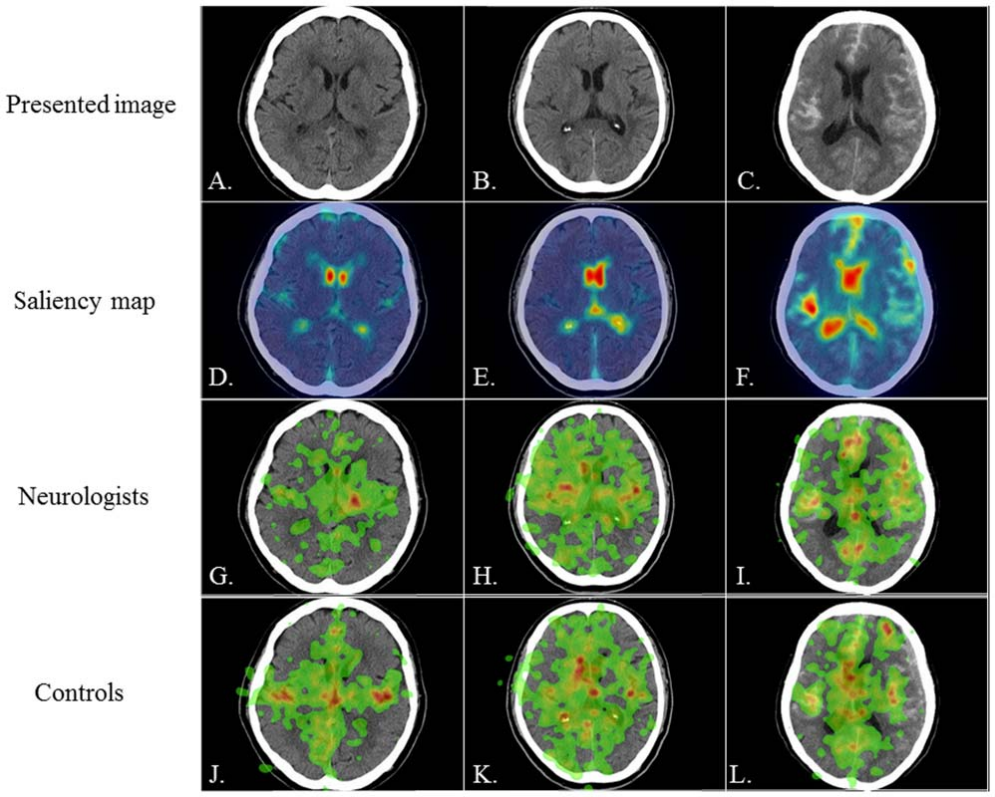
The presented images (images 4–6), saliency maps, and heat maps. A–C: the three CT images presented to subjects, D–F: saliency maps, G–I: heat maps in neurologists, J–L: heat maps in controls. Presented CT images are lacunar infarction (A: image 4), hyperacute cerebral infarction with old infarctions (B: image 5), and subarachnoid hemorrhage with acute subdural hemorrhage (C: image 6). Saliency maps reveal that the most outstanding areas are the ventricles (D, E, F). Heat maps reveal that neurologists gaze more frequently at the clinically important lesions than controls do in all images; these are the lacunar infarction area in image 4 (G), the hyperacute MCA infarction area in image 5 (H), and the acute subdural hemorrhagic area in image 6 (I).

We calculated the cumulative duration for which the subjects gazed at each pixel of each individual image. For descriptive purposes, heat maps, or graphical color-coded maps showing the distribution of eye-fixation positions, were created for each image using SR Research Data Viewer ver. 1.3.137. One heat map per image was created for each group, yielding a total of 12 heat maps (see Figures 1 and 2). To create a heat map, a two-dimensional Gaussian function was applied to each eye-fixation point. The Gaussian center was located at the eye-fixation position, the width of the Gaussian function was influenced by an adjustable sigma value (set at 0.8) in degrees of visual angle, and the height of the Gaussian function was weighted by the duration of individual eye-fixations. After the above process was applied to all eye-fixation points, these Gaussians were normalized and overlaid in a color-coded fashion onto the original image.

### ROI analysis

The outline of each ROI was extracted using the Intuos graphics tablet system (WACOM Co., Saitama, Japan), which gave the pixel positions of the ROI outline. The cumulative dwell time of eye-position in each ROI was plotted against the presentation time every 2.5 ms. The latency (seconds) for each lesion selected as an ROI was also measured in both groups.

### Statistical analysis

For the precise analysis of gaze patterns in two typical images (2 and 3), ROI analysis was conducted. Two ROIs were selected per image. The main lesion, which was looked at by both neurologists and controls, was selected as one ROI. The other ROI was the specific area in the heat map where the neurologists' gaze was most frequently focused, irrespective of its saliency. The dwell time at each ROI was analyzed using two-way analysis of variance (ANOVA) with repeated measures in one factor (within-subject factor: presentation time; between-subject factor, neurologists-controls). If necessary, the Greenhouse-Geisser correction was used to evaluate nonsphericity. The latency to ROI was analyzed using Mann-Whitney's U test or Fisher's exact probability test. *P* values of less than 0.05 were considered significant. Statistical analysis was performed using the SPSS software package (ver. 16.0; SPSS Inc., Chicago, Illinois, USA).

## Results

Figures 1 and 2 show the six images presented to subjects. A saliency map color-coded according to the strength of salience was overlaid onto each image. Higher salience areas are shown in red, intermediate areas in yellow, and lower salience areas in blue. Separately, a heat map color-coded according to the duration of eye-fixation was overlaid onto each image; areas attracting longer eye-fixations are shown in red, areas attracting intermediate-length eye-fixations in yellow, and areas attracting shorter eye-fixations in green.

### Image 1: normal brain

Figure 1D displays the saliency map of a normal brain CT image that was presented to subjects (Figure 1A). The most outstanding areas were the ventricles and cistern (red color) along the midline. Figures 1G and 1J display the heat maps for neurologists and controls, respectively. In both neurologists and controls, the eye-fixation positions were clustered frequently over the midline, especially in the ventricles and cistern on the midline (red color), which approximately coincided with the most outstanding areas in the saliency map (second row). On the other hand, the eye-fixation position of neurologists also extended widely to the bilateral parenchyma (green color). In other words, neurologists tended to gaze at the bilateral parenchyma, which has a low salience, more frequently than controls did.

### Image 2: cerebral hemorrhage from putamen

**Figure 3:**
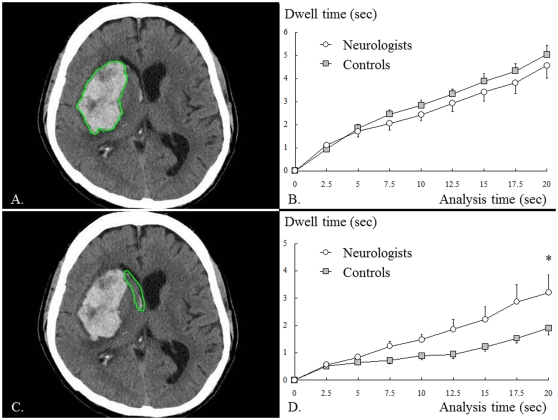
Cerebral hemorrhage from putamen (image 2). A: Region of interest (ROI) surrounding the large hemorrhagic area, B: dwell time in the ROI (A), C: ROI surrounding the intra-ventricular hemorrhage, D: dwell time in the ROI (C). The dwell time in the large hemorrhagic area did not significantly differ between the two groups (B), whereas the dwell time in the intra-ventricular hemorrhage was significantly longer for neurologists than for controls (D).

The brain CT image in Figure 1B shows cerebral hemorrhage at the right putamen. Figure 1E displays the saliency map for this image. The most outstanding area in the saliency map was the large hemorrhagic area (red color). The heat maps of both subject groups (Figures 1H, 1K) show that the eye-fixation positions were likewise focused in this large hemorrhagic area (red color), approximately coinciding with the most salient area. The dwell time over the ROI surrounding the large hemorrhagic area was comparable for the two groups (Figure 3A, 3B). ANOVA revealed that the two groups had no significant difference in dwell time (presentation time × subject group, F_1.939_ = 0.571, *P* = 0.564, and ε = 0.242; effect of subject group: F_1_ = 0.968, *P* = 0.336).

One major difference between the two groups with regard to this image involves the intra-ventricular hemorrhage along the midline (red color): unlike controls, neurologists tended to focus their gaze in this region as well as in the large hemorrhagic area, though the intra-ventricular hemorrhage is relatively inconspicuous in the saliency map (Figure 1E, 1H). When the intra-ventricular hemorrhage was selected as an ROI (Figure 3C, 3D), ANOVA revealed that the two groups significantly differed in their dwell time over this ROI (presentation time × subject group, F_1.391_ = 2.836, *P* = 0.092, and ε = 0.174; effect of subject group: F_1_ = 5.422, *P* = 0.030): neurologists' gaze stayed over this ROI significantly longer than controls' gaze did.

To summarize, dwell time in the large hemorrhagic area, the most outstanding area in the image, was not different between the two groups, but the dwell time in the intra-ventricular hemorrhage, a relatively inconspicuous area, was significantly longer in neurologists than in controls.

### Image 3: cerebral infarction due to embolism

**Figure 4:**
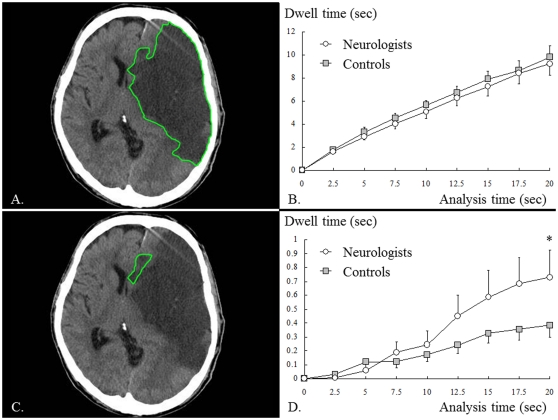
Cerebral infarction due to embolism (image 3). A: ROI surrounding the large infarction area, B: dwell time in the ROI (A), C: ROI surrounding the ACA infarction area, D: dwell time in the ROI (C). The dwell time in the large infarction area did not significantly differ between the two groups (B), whereas the dwell time in the ACA infarction area was significantly longer for neurologists than for controls (D).

The brain CT image in Figure 1C shows cerebral embolism with occlusion of the left internal carotid artery (ICA). According to the saliency map shown in Figure 1F, the most outstanding area was a region of physiological calcification due to aging (red color), which was, however, not of clinical importance in reading the brain CT image. The heat maps in neurologists and controls (Figures 1I, 1L) revealed that only control subjects focused their gaze on this region of physiological calcification (red color). In contrast, both groups similarly gazed at the large infarction area, which is of relatively low salience. Figure 4A displays the ROI surrounding the large infarction area and Figure 4B displays the dwell time. Throughout the entire presentation period, the dwell time over the large infarction area was similar between the two groups. ANOVA revealed that the two groups had no significant difference in dwell time (presentation time × subject group, F_1.546_ = 0.155, *P* = 0.803, and ε = 0.193; effect of subject group: F_1_ = 0.345, *P* = 0.563).

On the other hand, the gaze of neurologists was also focused in the infarction area fed by the anterior cerebral artery (ACA), which is clinically important though it is not salient (see Discussion). The ACA infarction area received more frequent eye-fixation and significantly longer dwell time from neurologists than from controls. As shown in Figures 4C and 4D, when the ACA infarction area within the large infarction area was selected as an ROI, ANOVA showed that the two groups significantly differed in dwell time (presentation time × subject group, F_1.324_ = 3.077, *P* = 0.080, and ε = 0.165; effect of subject group: F_1_ = 5.531, *P* = 0.028). Although the dwell time in the large infarction area was not different between the two groups, the dwell time in the ACA infarction area was significantly longer in neurologists than in controls.

### Images 4–6: masked lesions

The other brain CT images that were presented to subjects show lacunar infarction (Figure 2A: image 4), hyperacute cerebral infarction with old infarctions (Figure 2B: image 5), and subarachnoid hemorrhage with acute subdural hemorrhage (Figure 2C: image 6). The saliency maps for these images show that the most outstanding area was typically the ventricles, displayed in red on the saliency maps (Figures 2D, 2E, 2F). The heat maps for neurologists and controls revealed that, in all three of these images, neurologists gazed at masked (less conspicuous) cerebrovascular lesions that were nevertheless important for the diagnosis more often than controls did. In image 4, neurologists noticed the lacunar infarction area (red color), whereas controls gazed at the ventricle and cortical atrophy (Figure 2G, 2J). In image 5, neurologists gazed at the hemispheres asymmetrically, whereas controls gazed at both hemispheres equally. In addition, neurologists noticed the hyperacute right middle cerebral artery (MCA) infarction area where the border between the cortex and subcortical white matter and the outline of the basal ganglia were obscured (green color) (Figure 2H, 2K). In image 6, neurologists clearly noticed the left acute subdural hemorrhagic area (green color), whereas controls undoubtedly missed it (Figure 2I, 2L). In conclusion, neurologists were much more likely to notice masked lesions with low salience than controls were.

### Latency before gaze entered ROIs

In image 2, the median latency to the large hemorrhagic area was 0.5 seconds in neurologists and 0.6 seconds in controls (Mann-Whitney's U test, p = 0.463). In image 3, the median latency after which gaze entered the ACA infarction area was 11.5 seconds in neurologists, and was not obtained in controls because more than half of the control subjects missed it (the area was noticed by nine of 12 neurologists compared to only three of 12 controls). Therefore, neurologists noticed the ACA infarction area more frequently than controls did (Fisher's exact probability test, p = 0.039). The median latency to the intra-ventricular hemorrhage in image 2 and that to the large infarction area in image 3 were also unobtainable because the first eye-fixation point was already within the lesion.

## Discussion

Here we showed that neurologists and controls differ in the way they view brain CT images, although our controls had some knowledge about the brain. This study revealed the following findings: in image 2, both neurologists and controls (other medical practitioners) similarly gazed at high-salience areas such as the large hemorrhagic area. In image 3, however, controls gazed at the region of physiological calcification, which was highly salient but which lacked clinical importance for reading the brain CT image. Neurologists, in contrast, gazed at the ACA infarction area which was not exceptionally salient but which was clinically important. Similar findings were obtained for other images: only neurologists gazed often at low-salience areas with clinical importance such as the parenchyma (image 1), intra-ventricular hemorrhage (image 2), ACA infarction area (image 3) and masked lesions (images 4–6). This difference between the two groups in the tendency to gaze at less-salient clinically important areas was increasingly apparent with time: the dwell time in the ROIs began to differ at least 5 seconds after an image presentation (see ROI analyses for images 2 and 3). To summarize, both neurologists and controls tended to gaze at high-saliency areas, but neurologists gazed more frequently at areas that were less salient but clinically important.

Through saliency mapping, this study confirmed that two different types of visual attention, i.e., top-down instruction and bottom-up salience, are used in neurologists and controls when viewing brain CT images. Both neurologists and controls tended to gaze at high-salience areas which were not necessarily significant for interpreting the CT images, especially in the seconds immediately following an image presentation. Therefore, the attention of neurologists and controls is considered to be captured by visually salient objects, indicating that attentional deployment based on bottom-up salience is occurring in both groups. On the other hand, neurologists gazed more often at inconspicuous but clinically important areas outside the outstanding areas in the saliency maps. They also tended to look in areas where problems might be found: for example, the parenchyma often includes some lesions in cases such as lacunar infarction (image 1), intra-ventricular hemorrhage can induce non-communicating hydrocephalus (image 2), and an ACA infarction area can imply ICA occlusion (image 3). This indicates that neurologists actively directed attention to the collection of clinically important information regarding the diagnosis, cause, prognosis, and treatment of each case, information which is not necessarily associated with salience in CT images. Therefore the present findings suggest that, compared to control subjects, neurologists more effectively use the top-down instruction mode of visual attention, which is consistent with the importance of cognitive factors in active visual searching [15].

There have already been many papers on eye-tracking analysis during the reading of radiography results, including chest X-rays, mammography, pulmonary CT and dental CT [3], [16]–[22]. A holistic model has been proposed for the visual-search strategy employed by radiologists when reading mammograms [16], [17]. This model suggests that the initial detection of cancer on mammograms occurs before visual scanning, because even small cancers are usually detected by radiological experts within 1.0 second, a length of time which is too short to allow for lesion detection using central vision only. This model is also referred to as gestalt-like perception, and has been suggested as the means of recognition of familiar faces [23]–[25]. In other words, the visual-search strategy used by radiologists in interpreting mammograms may consist of a pattern of “look-detect-scan” rather than “scan-look-detect” [16]. However, we consider that the holistic “look-detect-scan” model is not always applicable to reading brain CT images, and that a search process more like a “scan-look-detect” pattern might be frequently used. In fact, neurologists often gazed at clinically important areas for over 1.0 second, and the median latency to the ACA infarction area was 11.5 seconds. This seems to be explained by the difference between the complexity of brain CT and the simplicity of mammography: the complexity of brain CT might contribute to the difficulty in using gestalt-like perception to interpret it.

To date, saliency mapping analysis has only been used with images of visual scenes [6], [8], [14], although this study showed that it is also applicable to radiographic brain CT images. In the future, saliency maps might be useful for addressing other interesting issues. For example, it might be interesting to compare the gaze of neurologists with that of radiologists. Because the two groups would consist of neuroradiologic diagnosis specialists, differing only in the area of their expertise, any difference in the gaze patterns might help identify the pattern of attentional deployment required for diagnostic processes. Alternatively, it might also be interesting to apply saliency mapping to brain CT images showing events other than cerebrovascular accidents, such as tumor, inflammation, and degeneration.

It is worth noting that saliency maps do not perfectly predict gaze direction related to bottom-up salience, because subjects practically never gazed at the rim of the cranium, which had the highest saliency of any area in the images. Therefore, fixation might not always be necessary for a human to identify the most salient objects in an image.

In conclusion, the analysis of saliency maps is applicable even for studying gaze behavior during the reading of brain CT images. While both neurologists and control subjects tend to look at visually salient positions, neurologists also intentionally scan areas of clinical importance in reading brain CT images showing cerebrovascular accidents. Thus both neurologists and control subjects use the “bottom-up salience” mode of visual attention, while neurologists more effectively use the “top-down instruction” mode.
